# Complete Genome Sequence of a Newcastle Disease Genotype XIII Virus Isolated from Indigenous Chickens in Zambia

**DOI:** 10.1128/genomeA.00841-17

**Published:** 2017-08-24

**Authors:** Celia Abolnik, Chrisborn Mubamba, George Dautu, Bruce Gummow

**Affiliations:** a Department of Production Animal Studies, Faculty of Veterinary Science, University of Pretoria, Pretoria, South Africa; b Discipline of Veterinary Science, College of Public Health, Medical and Veterinary Sciences, James Cook University, Queensland, Australia; c Department of Veterinary Services, Ministry of Fisheries and Livestock, Lusaka, Zambia

## Abstract

The first complete genome sequence of an African-origin Newcastle disease virus belonging to genotype XIII is described here. The virulent strain chicken/Zambia/Chiwoko/2015 was isolated from diseased chickens in 2015.

## GENOME ANNOUNCEMENT

Newcastle disease is a highly contagious and lethal disease of poultry and many other bird species caused by certain genotypes of avian paramyxovirus type 1 (aPMV-1). aPMV-1, or Newcastle disease virus, is classified as an avulavirus of the *Paramyxoviridae* family, with a single-stranded negative-sense RNA genome (1). The genome encodes six proteins: nucleocapsid protein (NP), phosphoprotein (P), matrix protein (M), fusion protein (F), hemagglutinin-neuraminidase protein (HN), and large polymerase protein (L). Two additional proteins, V and W, are produced during RNA editing of the P mRNA (2). F protein gene sequences are used to phylogenetically classify aPMV-1 strains into class I or class II, with 18 genotypes (I to XVIII) of mixed virulence in class II (3). Genotype XIII viruses are virulent in poultry, and the ancestral strain was recovered from a cockatoo in India in 1982 (3). Three subgenotypes, namely, XIIIa, XIIIb, and XIIIc, have been described (4). Subgenotype XIIIa strains have been isolated during poultry outbreaks in Europe, Africa, and the Middle East, but subgenotypes XIIIb and XIIIc remain localized to India and Pakistan (3, 4).

In Africa, genotype XIII strains have been recovered from an ostrich in South Africa in 1995, chickens in Burundi in 2008, and passerines in Tanzania in 2010 (5, 6), but only F protein sequences are available. We report here the first complete genome sequence for a genotype XIII virus isolated on the African continent. Strain chicken/Zambia/Chiwoko/2015 was isolated from samples collected from clinically sick indigenous chickens at Chiwoko, an area located 60 km southwest of Chipata city in eastern Zambia.

Virus isolation in 9-day-old specific-pathogen-free embryonated chicken eggs was performed at Deltamune Laboratory, Pretoria, South Africa, according to the OIE-recommended procedure (7). Three hundred nanograms of total RNA extracted from the alantoic fluid was used to generate a transcriptomic library using the recommended protocol from the TransPlex whole transcriptome amplification kit (Sigma-Aldrich, Johannesburg, South Africa). A High Pure PCR product purification kit (Roche Diagnostics, Mannheim, Germany) was used for purification of the amplification product. Nextera libraries were prepared and analyzed on an Illumina MiSeq apparatus by the sequencing service provider Inqaba Biotech (Pretoria, South Africa). Paired-end reads were trimmed for adapters and assembled in the CLC Genomics Workbench version 7.1.2 (Qiagen, Aarhus, Denmark). The genome of strain chicken/Zambia/Chiwoko/2015 is 15,192 nucleotides in length. This virus shares 95% nucleotide sequence with Indian strain chicken/Bareilly/01/2010 (GenBank accession no. KJ577585), classified as subgenotype XIIIa (4). The Chiwoko strain F protein cleavage site motif of _113_RRQKR↓F^117^ conforms with the definition of a virulent virus (7). Continuing Newcastle disease outbreaks in Africa and the strains involved must be monitored so that more effective control strategies may be implemented.

### Accession number(s).

The complete genome sequence of aPMV-1 strain chicken/Zambia/Chiwoko/2015 has been deposited in GenBank under the accession number MF409241.

## References

[1] Alexander DJ 2000 Newcastle disease and other avian paramyxoviruses. Rev Sci Tech 19:443–462. 10935273 10.20506/rst.19.2.1231

[2] Steward M, Vipond IB, Millar NS, Emmerson PT 1993 RNA editing in Newcastle disease virus. J Gen Virol 74:2539–2547. doi:10.1099/0022-1317-74-12-2539. 8277263

[3] Dimitrov KM, Ramey AM, Qiu X, Bahl J, Afonso CL 2016 Temporal, geographic, and host distribution of avian paramyxovirus 1 (Newcastle disease virus). Infect Genet Evol 39:22–34. doi:10.1016/j.meegid.2016.01.008. 26792710

[4] Das M, Kumar S 2017 Evidence of independent evolution of genotype XIII Newcastle disease viruses in India. Arch Virol 162:997–1007. doi:10.1007/s00705-016-3182-3. 28035479

[5] Cattoli G, Fusaro A, Monne I, Molia S, Le Menach A, Maregeya B, Nchare A, Bangana I, Maina AG, Koffi JN, Thiam H, Bezeid OE, Salviato A, Nisi R, Terregino C, Capua I 2010 Emergence of a new genetic lineage of Newcastle disease virus in West and Central Africa—implications for diagnosis and control. Vet Microbiol 142:168–176. doi:10.1016/j.vetmic.2009.09.063. 19939590

[6] Benson DA, Clark K, Karsch-Mizrachi I, Lipman DJ, Ostell J, Sayers EW 2015 GenBank. Nucleic Acids Res 43:D30–D35. doi:10.1093/nar/gku1216. 25414350 PMC4383990

[7] OIE 2015 Chapter 2.3.1.4. Newcastle disease (infection with Newcastle disease virus). Manual of diagnostic tests and vaccines for terrestrial animals. World Organisation for Animal Health, Paris, France http://www.oie.int/fileadmin/Home/eng/Health_standards/tahm/2.03.14_NEWCASTLE_DIS.pdf.

